# *In Vitro* Activity of Vancapticin MCC5145 against Methicillin-Resistant Staphylococcus aureus from Periprosthetic Joint Infection

**DOI:** 10.1128/AAC.02443-20

**Published:** 2021-04-19

**Authors:** Hye-Kyung Cho, Melissa J. Karau, Kerryl E. Greenwood-Quaintance, Karl A. Hansford, Matthew A. Cooper, Mark A. T. Blaskovich, Robin Patel

**Affiliations:** a Division of Clinical Microbiology, Department of Laboratory Medicine and Pathology, Mayo Clinic, Rochester, Minnesota, USA; b Centre for Superbug Solutions, Institute for Molecular Bioscience, The University of Queensland, St Lucia, Queensland, Australia; c Division of Infectious Diseases, Department of Medicine, Mayo Clinic, Rochester, Minnesota, USA

**Keywords:** lipoglycopeptides, methicillin-resistant *Staphylococcus aureus*, prosthesis-related infections

## LETTER

Methicillin-resistant *Staphylococcus aureus* (MRSA) periprosthetic joint infection (PJI) can be challenging to treat due to biofilm formation, alongside sometimes limited vancomycin activity (1–3). Vancapticins are semisynthetic vancomycin derivatives with membrane-targeting motifs added to the C terminus, resulting in enhanced affinity and avidity for membrane-bound lipid II, the vancomycin target (4, 5). Supplementation with 0.002% polysorbate 80 (P-80) is recommended to prevent adherence to plastic surfaces when determining MICs of the lipoglycopeptides telavancin, dalbavancin, and oritavancin (6, 7). Vancapticins, which have structures similar to those of other lipoglycopeptides, are positively charged and adhere to plastic surfaces, thereby hypothetically benefitting from the addition of P-80, with similar improvements in MICs obtained using nonbinding plates (8).

Vancapticin MCC5145 MICs of 37 PJI-associated MRSA isolates collected from 2000 to 2016 were determined using broth microdilution with and without P-80 (6, 7). Minimum biofilm inhibitory concentrations (MBICs) and minimum biofilm bactericidal concentrations (MBBCs) were determined as described previously (9) (Table 1). Median MIC, MBIC, and MBBC values were 8-, 8-, and 4-fold lower, respectively, when supplemented with versus without P-80. Results were compared to those previously determined using the same isolates for vancomycin, dalbavancin, and oritavancin, except that two isolates were excluded from comparative analysis to vancomycin and dalbavancin (9–11). The MIC_90_ of 0.12 μg/ml (with P-80) was comparable to those of dalbavancin and oritavancin (0.06 and 0.12 μg/ml, respectively) and lower than that of vancomycin (2 μg/ml) (9–11). The MBIC_90_ of 0.12 μg/ml (with P-80) was comparable to that of dalbavancin (0.25 μg/ml) (10) and lower than those of oritavancin and vancomycin (both 2 μg/ml) (9, 11). The MBBC_90_ (with P-80) of 2 μg/ml was comparable to those of dalbavancin and oritavancin (2 and 4 μg/ml) (9, 10) and lower than that of vancomycin (>128 μg/ml) (11).

**TABLE 1:** MCC5145 MIC, MBIC, and MBBC of methicillin-resistant *Staphylococcus aureus* (*n* = 37)

Inhibitory or bactericidal concn type and test agent(s)	No. of isolates (cumulative percentage) with MIC, MBIC, or MBBC at concn [μg/ml (%)] of:										MIC_50_, MBIC_50_, or MBBC_50_ (μg/ml)	MIC_90_, MBIC_90_, or MBBC_90_ (μg/ml)
	0.015	0.03	0.06	0.12	0.25	0.5	1	2	4	8		
MIC
MCC5145 without P-80	18 (48.6)	17 (94.6)	2 (100)	0.5	0.5
MCC5145 with P-80	3 (8.1)	1 (10.8)	27 (83.8)	6 (100)	0.06	0.12
MBIC
MCC5145 without P-80	5 (13.5)	21 (70.3)	9 (94.6)	2 (100)	0.5	1
MCC5145 with P-80	1 (2.7)	19 (54.1)	17 (100)	0.06	0.12
MBBC
MCC5145 without P-80	1 (2.7)	19 (54.1)	9 (78.4)	8 (100)	2	8
MCC5145 with P-80	2 (5.4)	11 (35.1)	15 (75.7)	8 (97.3)	1 (100)	1	2

When comparing the MCC5145 and vancomycin susceptibility of three quality control strains with or without P-80, MCC5145 MICs, MBICs, and MBBCs without P-80 were 4- to 64-, 2- to 16-, and 2- to 4-fold higher, respectively, than those with P-80, whereas vancomycin showed similar values with or without P-80 (Table 2).

**TABLE 2:** MCC5145 and vancomycin MIC, MBIC, and MBBC of three quality control Staphylococcus aureus strains with and without P-80

Strain	MCC5145						Vancomycin					
	MIC (μg/ml)		MBIC (μg/ml)		MBBC (μg/ml)		MIC (μg/ml)		MBIC (μg/ml)		MBBC (μg/ml)	
	+P80	−P80	+P80	−P80	+P80	−P80	+P80	−P80	+P80	−P80	+P80	−P80
ATCC 43300 (methicillin resistant)	0.06	0.25	0.06	0.5	2	4	2	2	1	2	128	>128
ATCC 29213 (methicillin susceptible)	0.015	1	0.06	1	0.5	2	2	1	1	2	128	>128
ATCC 25923 (methicillin susceptible)	0.06	0.5	1	2	4	8	2	2	2	8	16	32

**FIG 1:**
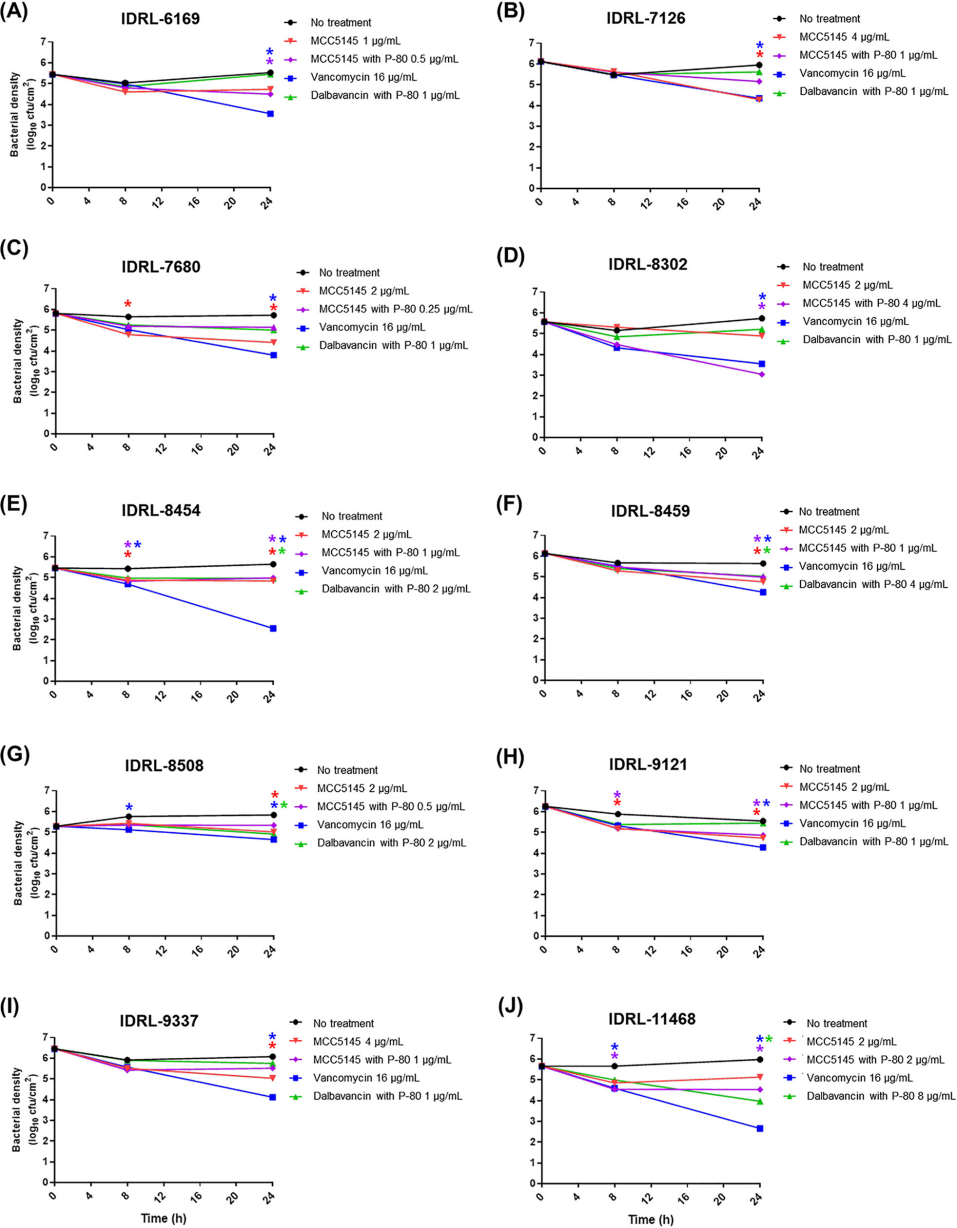
Biofilm time-kill curves of 10 methicillin-resistant Staphylococcus aureus isolates. (A) IDRL-6169, (B) IDRL-7126, (C) IDRL-7680, (D) IDRL-8302, (E) IDRL-8454, (F) IDRL-8459, (G) IDRL-8508, (H) IDRL-9121, (I) IDRL-9337, and (J) IDRL-11468. All isolates were tested with MCC5145 with and without P-80 and dalbavancin with P-80 at 1× MBBC, and with vancomycin at the *fC_max_*. *, *P* < 0.05 compared with the no treatment group at each time point by two-way analysis of variance with Tukey’s multiple-comparison test. Data presented are means (*n* = 3).

Biofilm time-kill assays were performed as previously described (12) using 10 PJI isolates (Table 3). Biofilms on Teflon coupons were treated with 1× MBBC for dalbavancin and MCC5145 and *fC_max_* (free plasma concentration) for vancomycin (16 μg/ml [13]). MCC5145 reduced biofilms of 3 of 10 isolates after 8 h and 7 of 10 after 24 h compared with controls (Fig. 1). MCC5145 with P-80 reduced biofilms of 3 of 10 isolates after 8 h and 6 of 10 after 24 h compared with controls. Vancomycin reduced biofilms of 3 of 10 isolates after 8 h and all 10 isolates after 24 h compared with controls. Dalbavancin with P-80 did not reduce biofilms after 8 h for any isolate; however, there was a reduction after 24 h for 4 of 10 isolates compared with controls. Bactericidal activity, defined as ≥3-log_10_ CFU/cm^2^ reduction between 0 and 24 h (12), was not observed after 8 or 24 h for MCC5145, MCC5145 with P-80, vancomycin, or dalbavancin with P-80.

**TABLE 3:** MIC and MBBC values of each antimicrobial agent for 10 methicillin-resistant *Staphylococcus aureus* isolates

Isolate	MIC (μg/ml)				MBBC (μg/ml)			
	MCC5145	MCC5145 with P-80	Vancomycin^*a*^	Dalbavancin with P-80^*b*^	MCC5145	MCC5145 with P-80	Vancomycin^*a*^	Dalbavancinwith P-80^*b*^
IDRL-6169	0.25	0.015	1	0.03	1	0.5	>128	1
IDRL-7126	0.25	0.06	1	0.03	4	1	>128	1
IDRL-7680	0.25	0.06	2	0.03	2	0.25	>128	1
IDRL-8302	0.5	0.06	2	0.03	2	4	>128	1
IDRL-8454	0.5	0.06	1	0.03	2	1	>128	2
IDRL-8459	0.25	0.06	1	0.06	2	1	>128	4
IDRL-8508	0.25	0.06	1	0.03	2	0.5	>128	2
IDRL-9121	0.25	0.06	1	0.03	2	1	>128	1
IDRL-9337	0.5	0.06	1	0.25	4	1	>128	1
IDRL-11468	0.25	0.06	2	0.06	2	2	>128	8

a Vancomycin MIC and MBBC values are from a previous study (11), except for those for IDRL-11468; the MIC and MBBC of IDRL-11468 were tested in this study.

b Dalbavancin with P-80 MIC and MBBC values are from a previous study (10), except for those for IDRL-11468; the MIC and MBBC of IDRL-11468 were tested in this study.

Vancapticin MCC5145 has promising *in vitro* activity against PJI-associated MRSA but was not bactericidal against biofilms on Teflon. The addition of P-80 decreased MCC5145 MICs, MBICs, and MBBCs.
